# Contribution to the study of acoustic communication in two Belgian river bullheads (*Cottus rhenanus and C. perifretum*) with further insight into the sound-producing mechanism

**DOI:** 10.1186/1742-9994-10-71

**Published:** 2013-11-19

**Authors:** Orphal Colleye, Michael Ovidio, André Salmon, Eric Parmentier

**Affiliations:** 1 Laboratory of Functional and Evolutionary Morphology, University of Liège, Liège 4000, Belgium; 2 Biology of Behaviour Unit, Laboratory of Fish Demography and Hydroecology, University of Liège, Tihange 4500, Belgium

**Keywords:** Cottidae, Freshwater sculpins, Sound production, Hearing abilities, Morphological traits

## Abstract

**Background:**

The freshwater sculpins (genus *Cottus*) are small, bottom-living fishes widely distributed in North America and Europe*.* The taxonomy of European species has remained unresolved for a long time due to the overlap of morphological characters. Sound production has already been documented in some cottid representatives, with sounds being involved in courtship and agonistic interactions. Although the movements associated with sound production have been observed, the underlying mechanism remains incomplete. Here, we focus on two closely related species from Belgium: *C. rhenanus* and *C. perifretum*. This study aims 1) to record and to compare acoustic communication in both species, 2) to give further insight into the sound-producing mechanism and 3) to look for new morphological traits allowing species differentiation.

**Results:**

Both *Cottus* species produce multiple-pulsed agonistic sounds using a similar acoustic pattern: the first interpulse duration is always longer, making the first pulse unit distinct from the others. Recording sound production and hearing abilities showed a clear relationship between the sound spectra and auditory thresholds in both species: the peak frequencies of calls are around 150 Hz, which corresponds to their best hearing sensitivity. However, it appears that these fishes could not hear acoustic signals produced by conspecifics in their noisy habitat considering their hearing threshold expressed as sound pressure (~ 125 dB re 1 μPa). High-speed video recordings highlighted that each sound is produced during a complete back and forth movement of the pectoral girdle.

**Conclusions:**

Both *Cottus* species use an acoustic pattern that remained conserved during species diversification. Surprisingly, calls do not seem to have a communicative function. On the other hand, fish could detect substrate vibrations resulting from movements carried out during sound production. Similarities in temporal and spectral characteristics also suggest that both species share a common sound-producing mechanism, likely based on pectoral girdle vibrations. From a morphological point of view, only the shape of the spinelike scales covering the body allows species differentiation.

## Background

Cottidae is a family of demersal fish with about 300 species divided into 70 genera that are mostly marine and found in shallow coastal waters in the northern regions. There are also some freshwater representatives that inhabit lakes of North America and the main rivers in Northern Europe [1]. Within this family, sculpins or river bullheads (genus *Cottus*) are small, bottom-living freshwater fishes including many species widely distributed in North America and Europe. These fishes are stenotopic, inhabiting cold and well-oxygenated streams. Within their range, they have a very patchy distribution because their ecological requirements do not enable them to disperse over long distances [2,3]. Their reproductive behavior has been well studied. Males of most *Cottus* species excavate nest cavities under large rocks in which females enter and turn over, sticking the eggs to the nest roof. Males guard eggs until hatching [4,5]. *Cottus* species are well known for the intraspecific variability of some of their characters (colour pattern, fins shape, prickling), which made species differentiation difficult and resulted in gathering most of them in unresolved species complexes. For example, the European species were retained in a taxonomically unresolved “*Cottus gobio* complex” [6]. Recently, the taxonomy was revised based on molecular data and the review of morphological characters [3]. To date, there exists 15 diagnosable species of *Cottus* distributed in Europe: *C. gobio*, *C. hispaniolensis*, *C. rondeleti*, *C. petiti*, *C. aturi*, *C. duranii*, *C. perifretum*, *C. rhenanus*, *C. microstomus*, *C. koshewnikowi*, *C. transsilvaniae*, *C. haemusi*, *C. metae*, *C. scaturigo* and *C. poecilopus*.

Sound production has been documented in many representatives of the family Cottidae. Marine species from the North American Atlantic coast such as *Myoxocephalus scorpius* and *M. octodecimspinosus* are known to produce sounds [7-9]. Some freshwater representatives are also known to be soniferous. Ladich [10,11] highlighted that both sexes of the European river bullhead *Cottus gobio* produce knocking sounds while displaying threat postures during conspecific territorial defense. In North America, Whang & Janssen [12] found that the mottled sculpin *C. bairdi* produces knocking sounds associated with courtship, and Kierl & Johnston [13] reported sound production in the pygmy sculpin *C. paulus* during courtship and agonistic behaviors. All these observations imply that the role of sound production among cottids is an important part of their life history and retained through time [13]. Indeed, the recent phylogeny of the genus *Cottus* highlighted that *C. gobio* occupies a more basal or ancestral position than *C. paulus* and *C. bairdii* which are more derived species [14]. This suggests that sound production is a basal trait and will most likely be found in other *Cottus* species [13]. Besides sound production, there exist a few studies dealing with hearing sensitivities in Cottidae. Enger [15] reported that the peak frequency of the signal is within the auditory frequency range in *M. scorpius*. Coombs & Janssen [16] showed that the acoustic signal is in the effective frequency range of the lateral line in the mottled sculpin *C. bairdi*. Sculpins have no swimbladder so they are not able to detect pressure oscillations [17]. They can hear only via vibrations that move the body and cause the hair cells of the inner ear to move with the body relative to the dense otoliths suspended on their kinocilia [18,19]. It has also been shown that the mottled sculpin *C. bairdi* is able to locate substrate vibrations with both the ear and lateral line [20].

Barber & Mowbray [9] first studied the sound-producing mechanism in *M. octodecimspinosus* and stated that contractions of deep cranioclavicular muscles produce sounds during pectoral girdle movements. A characteristic head nodding movement and rapid adduction of the pectoral girdle relative to the skull have been observed during sound production in *C. gobio*[10] and *C. bairdi*[12], suggesting the swift movements observed during the head nod were similar to the mechanism of sound production in *M. octodecimspinosus*[10]. A forward jerking motion was observed during knock trains produced at high levels of motivation in *C. paulus*[13]. All these similarities in the mechanism lead to the conclusion that the family Cottidae shares a characteristic sound-producing mechanism [10]. However, there is still a need to go further into the understanding of the sound-producing mechanism in *Cottus* species, especially regarding the anatomical structure(s) responsible for sound production.

Here, we focus on two closely related *Cottus* species distributed in Belgium: *C. perifretum* is located in the Scheldt river while *C. rhenanus* occurs in Meuse drainages. Although they inhabit distinct rivers, they may occur in sympatry in the lower Rhine drainage, but not in syntopy [3]. *Cottus rhenanus* is found in the headwaters whereas *C. perifretum* inhabits the main rivers and the lower courses of the main tributaries. However, these species are not completely non-syntopic because they form very narrow hybrid zones through which there is apparently no or only very limited gene flow [21]. Although both species can be distinguished using molecular markers [22], it is quite tricky to identify them based only on morphological characters due to numerous overlap [3].

In this context, the present study aims to record and to compare sound production and hearing abilities in these two sculpin species in order to understand how acoustic communication evolved during species diversification. The purpose is also to give further insight into the sound-producing mechanism using a multidisciplinary approach that combines morphology, high-speed videos and sound analysis. To a lesser extent, it is also interesting to look for new morphological characters allowing the differentiation of both species.

## Results

### Sound production

**Figure 1:**
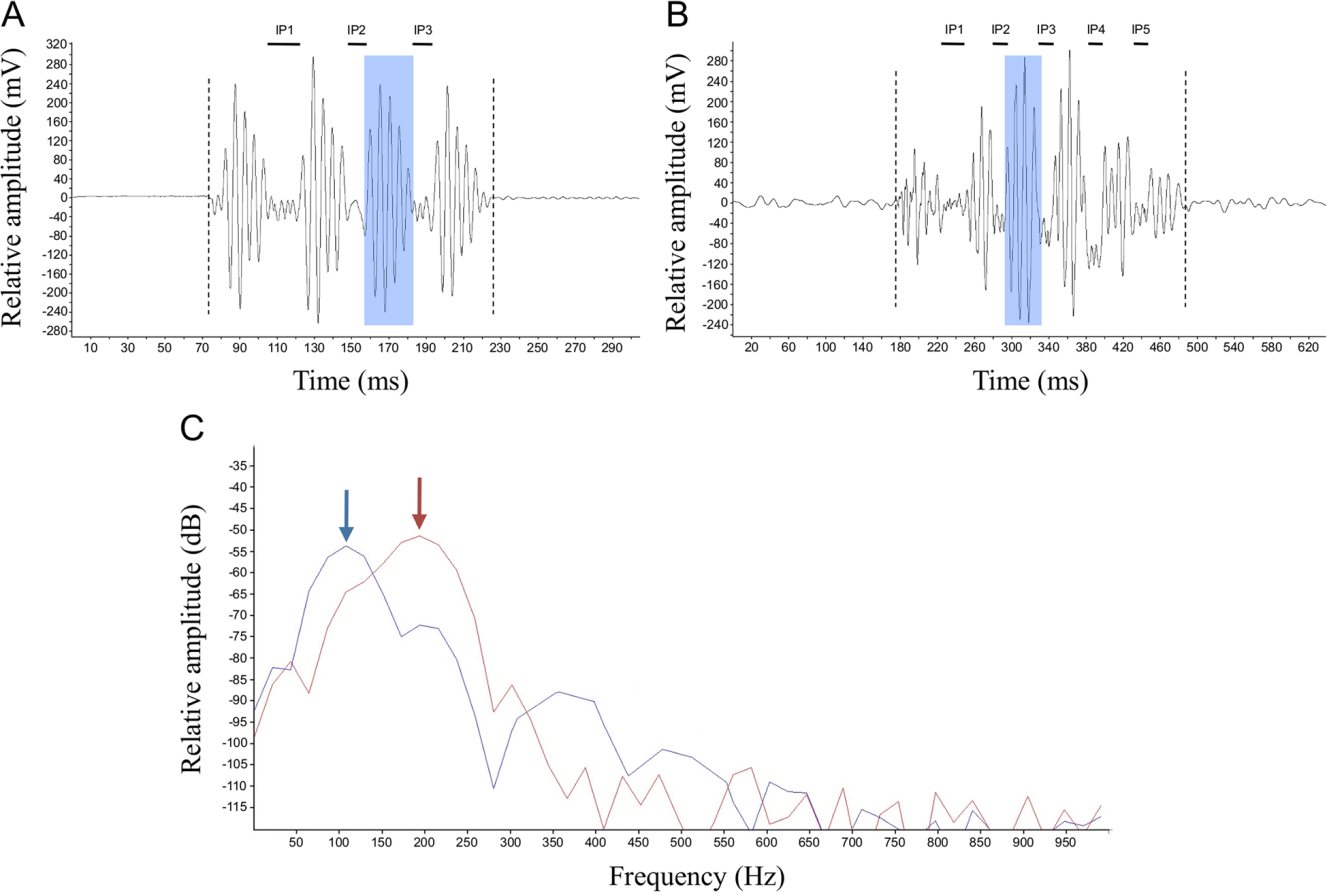
**Waveform of agonistic sounds produced by *Cottus rhenanus *and *C. perifretum*.** Oscillograms of multiple-pulsed sounds produced by *C. rhenanus***(A)** and *C. perifretum***(B)**. The vertical dotted lines delimit the sound duration, and the blue zones highlight a pulse unit in a series. The black bars correspond to the different interpulse durations in a sound. IP = interpulse. **(C)** Power spectra of sounds produced by *C. rhenanus* (red line) and *C. perifretum* (blue line) showing the difference in peak frequencies (see arrows).

All sounds were produced during agonistic encounters, which usually occurred when a fish approached the territory (i.e. halved flower pot) of a conspecific. Sounds were emitted during threatening and chasing. Multiple-pulsed sounds were observed when opponents got closer during long agonistic encounters. In both species, sounds were composed of short pulse units (< 40 ms) that can be emitted alone or in series, and in a narrow band of low frequencies (< 1 kHz) (Figure 1). Interpulse duration ranged from 8 to 23 ms in *C. rhenanus* and from 11 to 33 ms in *C. perifretum*. Number of pulses per sound varied from 2 to 4 pulses in *C. rhenanus* and from 3 to 6 pulses in *C. perifretum*.

The comparison between both species using Mann–Whitney U test revealed that means were significantly different for all acoustics features measured. Indeed, several acoustic features were greater in *C. perifretum* than in *C. rhenanus* (Figure 1, Table 1): sound duration (229.8 ± 41.5 *vs* 128.1 ± 21.3 ms, *U* = 2000, *P* < 0.0001), pulse duration (38.5 ± 7.6 *vs* 30.3 ± 3.9 ms, *U* = 3094, *P* < 0.0001), interpulse duration (19.2 ± 4.9 *vs* 14.3 ± 3.2 ms, *U* = 2220, *P* < 0.0001) and the number of pulses per sound (4.0 ± 0.9 *vs* 3.2 ± 0.5, *U* = 388, *P* < 0.0001). Inversely, dominant frequency (169.4 ± 23.7 *vs* 107.6 ± 18.8 Hz, *U* = 696.5, *P* < 0.0001) was higher in *C. rhenanus* than in *C. perifretum* (Figure 1C, Table 1).

**Table 1:** **Summary of the acoustic variables recorded in *Cottus rhenanus *** **and *C*. *perifretum***

**Sonic characteristics**	***Cottus rhenanus***			***Cottus perifretum***			
	**Mean ± S.D.**	**Ranges**	***n***	**Mean ± S.D.**	**Ranges**	***n***	***p*-value**
Sound duration (ms)	128.1 ± 21.3	85 – 175	84	229.8 ± 41.5	175 – 319	78	<0.0001
Pulse duration (ms)	30.3 ± 3.9	24 – 45	268	38.5 ± 7.6	22 – 87	318	<0.0001
Dominant frequency (Hz)	169.4 ± 23.7	110 – 237	268	107.6 ± 18.8	65 – 194	318	<0.0001
Interpulse duration (ms)	14.3 ± 3.2	8 – 24	184	19.2 ± 4.9	11 – 34	240	<0.0001
Number of pulses per sound	3.2 ± 0.5	2 – 4	84	4.0 ± 0.9	3 – 6	78	<0.0001

*p*-values refer to the results of the non-parametric Mann–Whitney U test. n, number of data analyzed.

**Figure 2:**
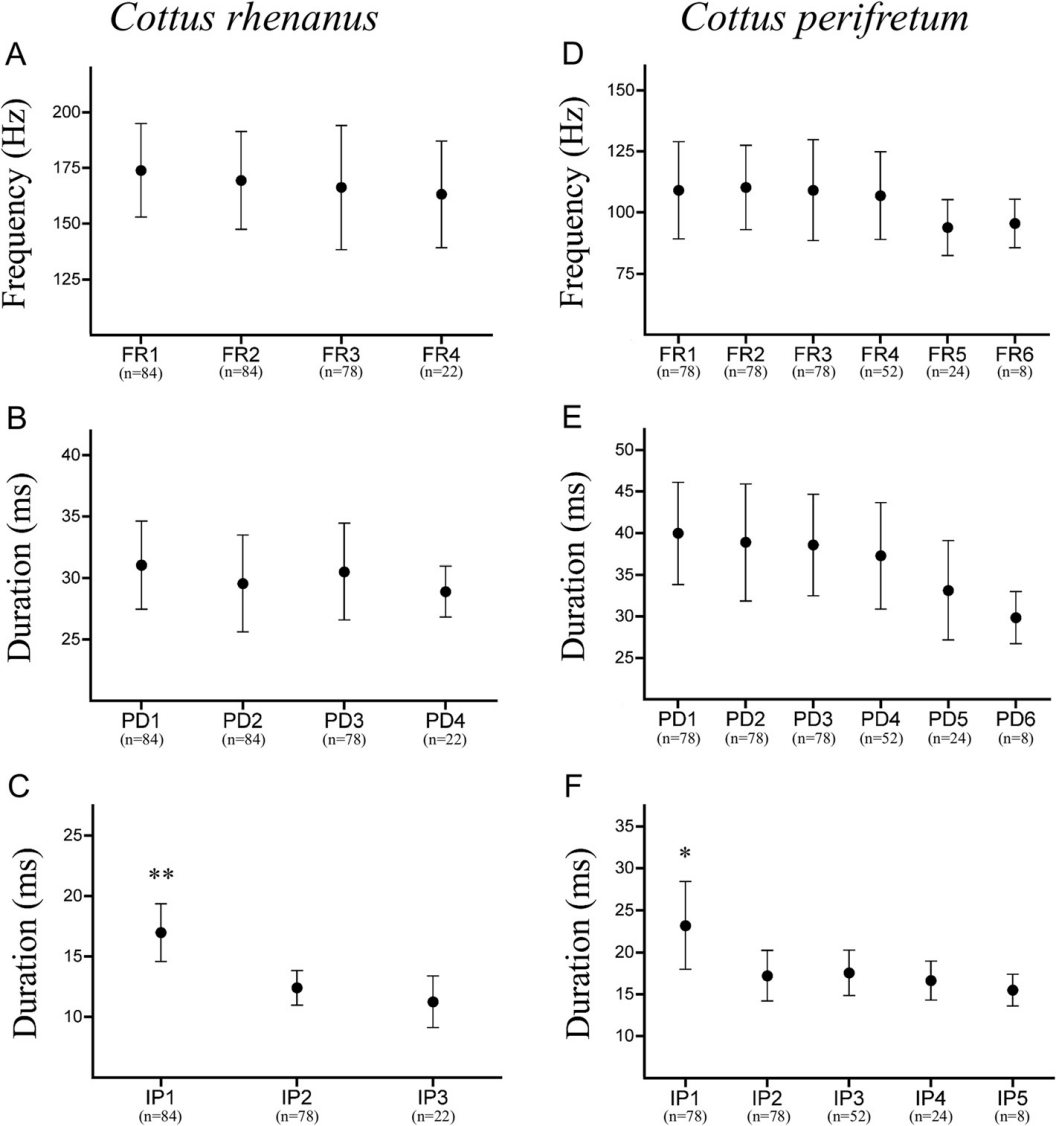
**Variation of acoustic features in agonistic sounds.** Comparisons of dominant frequencies **(A)**, pulse durations **(B)** and interpulse durations **(C)** within multiple-pulsed sounds produced by *Cottus rhenanus*. Same comparisons have been made with dominant frequencies **(D)**, pulse durations **(E)** and interpulse durations **(F)** within multiple-pulsed sounds produced by *C. perifretum*. FR = frequency; PD = pulse duration; IP = interpulse. Results are expressed as mean ± S.D. values. *Statistically significant according to Friedman’s test (*, p < 0,01; **, p < 0,001). n, number of data analyzed.

Further analysis of multiple-pulsed sounds highlighted that both species exhibited a similar acoustic pattern. Within each species, the first interpulse duration was always significantly longer than the others (*C. rhenanus*: *χ*^2^ = 20.46, d.f. = 2, *P <* 0.0001, Figure 2C – *C. perifretum*: *χ*^2^ = 14.93, d.f. = 4, *P* = 0.0048; Figure 2F), whereas no difference was observed among pulse durations (*C. rhenanus*: *χ*^2^ = 1.374, d.f. = 3, *P =* 0.7117, Figure 2B – *C. perifretum*: *χ*^2^ = 8.445, d.f. = 5, *P* = 0.1334, Figure 2E) and dominant frequencies (*C. rhenanus*: *χ*^2^ = 1.339, d.f. = 3, *P =* 0.7198; Figure 2A – *C. perifretum*: *χ*^2^ = 3.852, d.f. = 5, *P* = 0.5708, Figure 2D).

### Auditory sensitivities

**Figure 3:**
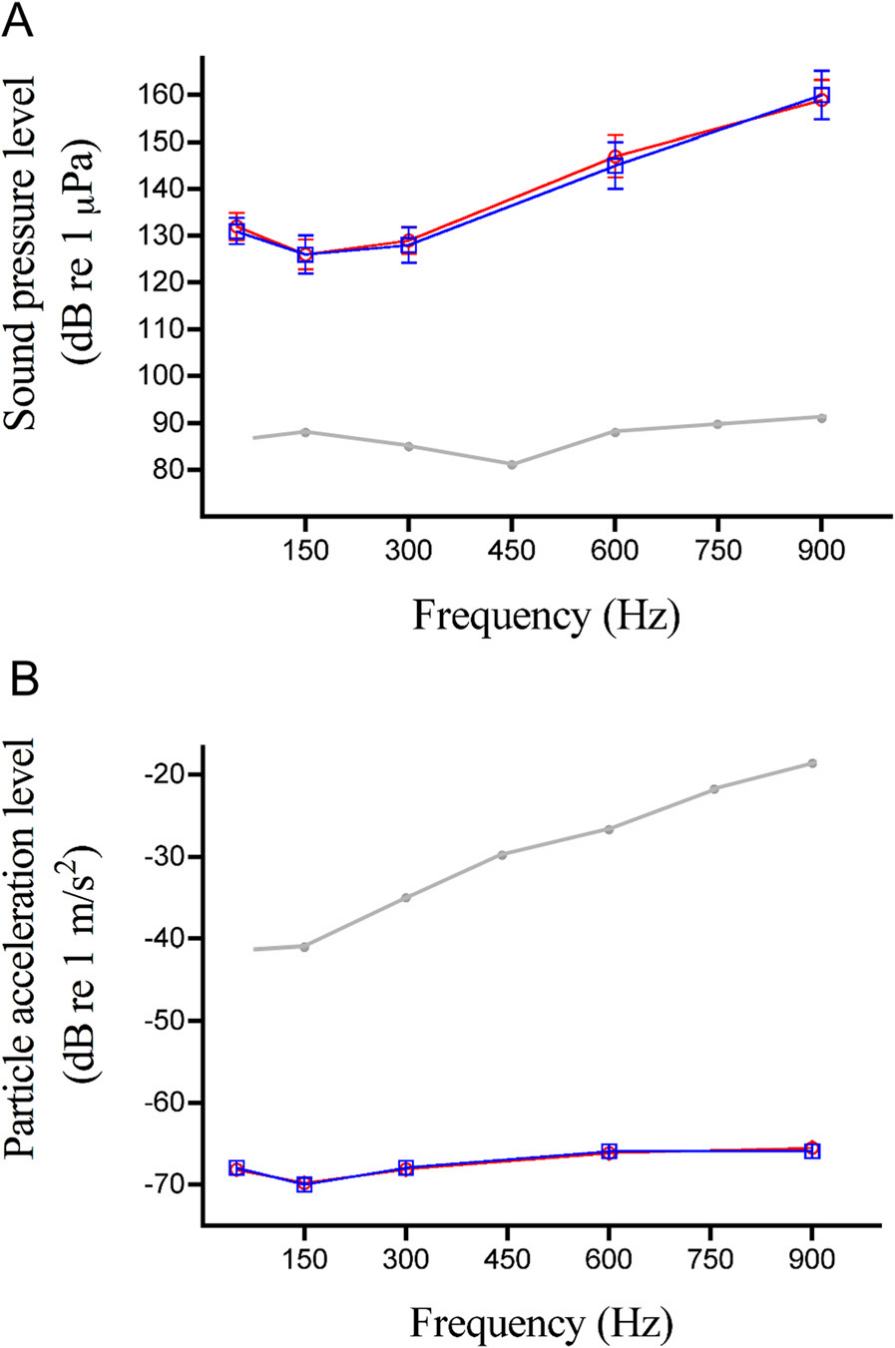
**Hearing thresholds of the two cottid species investigated. (A)** Sound pressure level (SPL) and **(B)** Particle acceleration level (PAL) audiograms showing auditory capacities measured in *Cottus rhenanus* (n = 11, blue line) and *C. perifretum* (n = 10, red line). Results are expressed as mean ± S.D. values. Grey lines represent hearing thresholds for the goldfish *Carassius auratus*[23]. Note that PALs are expressed in dB re 1 m s^-2^ to be compared to data from Radford et al. [23].

Both species exhibited clear responses to sound frequencies ranging from 50 to 900 Hz, whereas no response was detectable at 1200 Hz (Figure 3, Table 2). Auditory sensitivities did not significantly differ between species at any frequency tested (*P* > 0.05, Table 2). Indeed, audiograms were similarly shaped with their best hearing sensitivity at 150 Hz regardless of whether the threshold was expressed in terms of Sound Pressure Levels (SPLs) or Particle Acceleration levels (PALs) (Figure 3). Then, sensitivity showed a steep drop-off at lower and higher frequencies.

**Table 2:** Mean (± S.D.) hearing thresholds of the two cottid species investigated

	***Cottus rhenanus *** **(n = 11)**		***Cottus perifretum *** **(n = 10)**		
**Frequency**	**SPL threshold**	**PAL threshold**	**SPL threshold**	**PAL threshold**	***p*-value**
**(Hz)**	**(dB re 1 μPa)**	**(dB re 1** **μm/s**^**2**^**)**	**(dB re 1 μmPa)**	**(dB re 1** **μm/s**^**2**^**)**	
50	131 ± 2.8	52.0 ± 0.11	132 ± 2.9	51.9 ± 0.12	>0.05
150	126 ± 4.1	50.6 ± 0.18	126 ± 3.2	50.7 ± 0.16	>0.05
300	128 ± 3.8	52.1 ± 0.19	129 ± 2.9	52.0 ± 0.10	>0.05
600	145 ± 5.0	53.9 ± 0.10	147 ± 4.5	53.9 ± 0.07	>0.05
900	160 ± 5.2	54.1 ± 0.08	159 ± 4.2	54.0 ± 0.09	>0.05
1200	nr	nr	nr	nr	nr

n, number of specimens analyzed; nr, no response.

*p*-values refer to the results of the non-parametric Mann–Whitney U test.

PAL thresholds are determined from the *z*-axis component as the two horizontal axes (*x*- and *y*-axes) yielded much smaller particles accelerations (see Table 3).

Due to the vertical speaker axis, the vertical component (*z*-axis) of particle acceleration was used for plotting the PAL audiograms because it yielded much greater amplitudes than the two horizontal axes (*x*- and *y*-axes) at each frequency (Table 3).

**Table 3:** Particle accelerations in the three Cartesian directions measured at threshold levels at each test frequency

**Frequency**	***x*-axis *a***	***y*-axis *a***	***z*-axis *a***	**Mag *a***	**PAL**
**(Hz)**	**(μm/s**^**2**^**)**	**(μm/s**^**2**^**)**	**(μm/s**^**2**^**)**	**(μm/s**^**2**^**)**	**(dB re 1 μm/s**^**2**^**)**
50	128	118	358	398	51.99
150	124	116	293	338	50.58
300	121	105	372	405	52.15
600	121	113	471	498	53.94
900	145	137	462	503	54.03

Data show that most of the acoustic energy was along the *z*-axis, which is equivalent to the direct path (straight line from the transducer to the fish in the test tube). The *x*-axis is the rostrocaudal axis, and the *y*-axis is the lateral axis. *a*, particle acceleration; Mag *a*, magnitude acceleration of the three directions combined (e.g. [24,25]). The magnitude was calculated using the following equation: $\sqrt{\left(x^{2}+y^{2}+z^{2}\right)}$. The accelerations were then log transformed to convert to particle acceleration levels (dB re 1 μm/s^2^).

### Fish movements during sound production

High-speed video recordings highlighted that fish produced sounds by performing characteristic body movements, especially located at the level of the head and pectoral girdle. These movements occurred when the soniferous fish rested at the bottom on its pelvic fins, and raised its dorsal and pectoral fins with the mouth barely opened. Overall, each pulse unit was always accompanied by a series of movements including a forward motion of the pectoral girdle that took place simultaneously with the abduction of the opercular bones and suspensoria (i.e. cheek region), followed by a lowering of the neurocranium. The pectoral girdle, opercular bones and suspensoria immediately returned to their original position by performing reverse movements, whereas the neurocranium did not. Indeed, the neurocranium returned to its original position only after the last pulse unit had been emitted in the case of a multiple-pulsed sound. An additional movie file shows this in more detail (see Additional file 1). The time necessary to complete one back and forth cycle in *C. perifretum* (36 ± 11 ms, n = 8) is not significantly different from the pulse durations (*P* > 0.05, Table 1), confirming the relationship between these two events. Although it was not demonstrated using high-speed video, the sound-producing mechanism observed in *C. rhenanus* displayed exactly the same patterns (pers. obs.).

### Functional morphology

**Figure 4:**
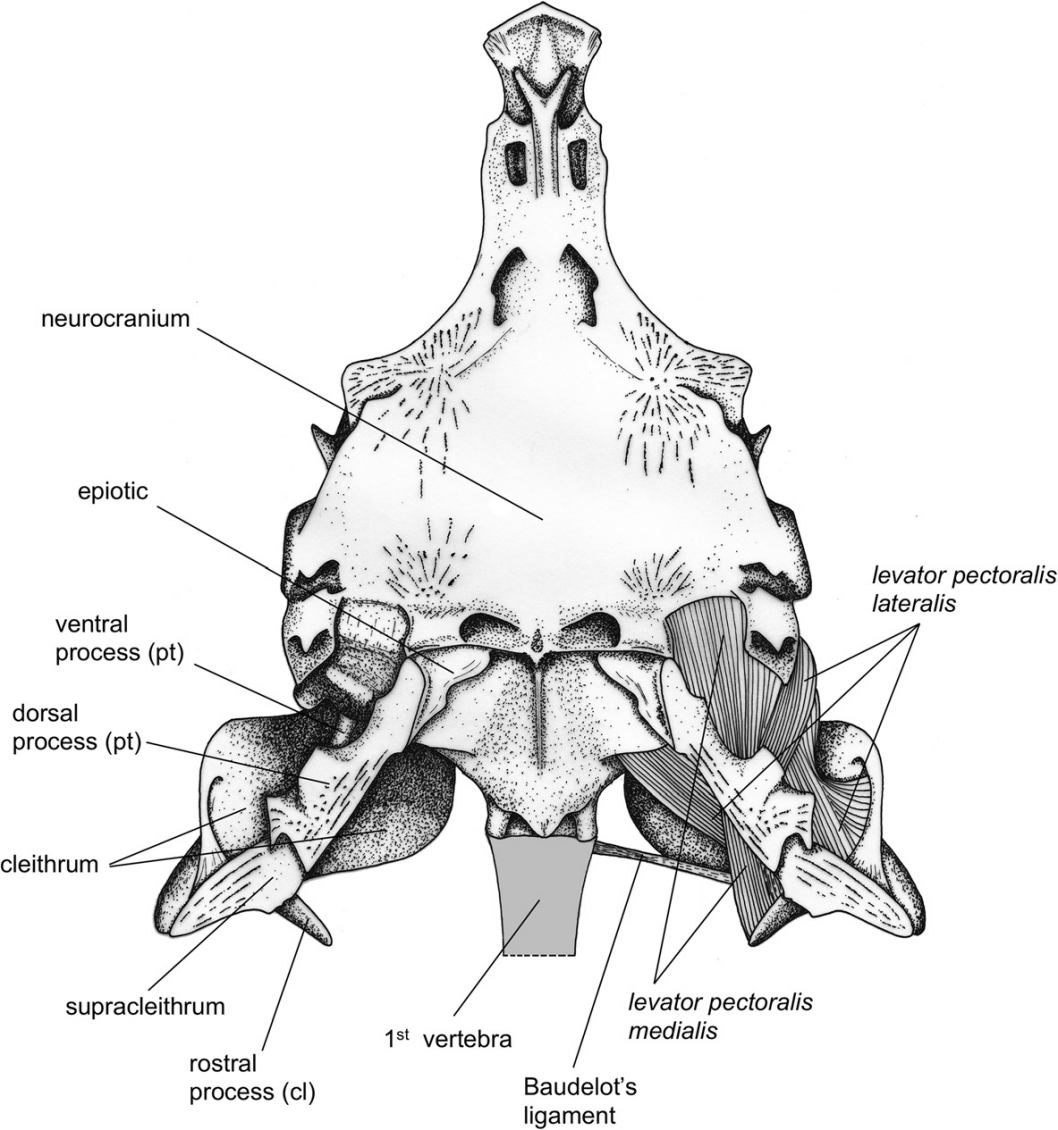
**Morphology of the neurocranium and pectoral girdle in *Cottus perifretum*.** Dorsal view of the skeletal elements of the pectoral girdle (left) and the different muscles involved in pectoral girdle adduction (right). pt, post-temporal; cl, cleithrum.

**Figure 5:**
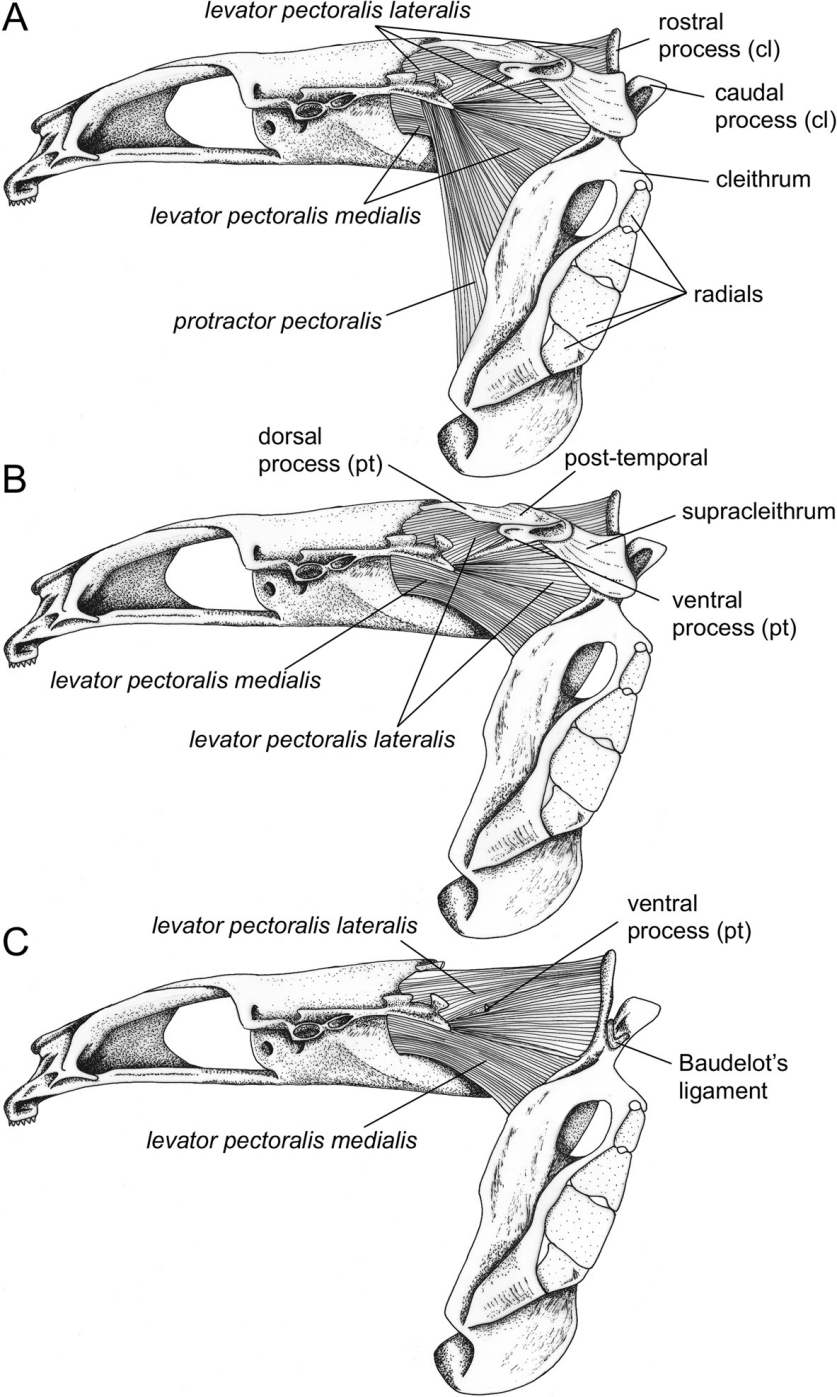
**Morphology of hard and soft tissues involved in sound production in *Cottus perifretum*.** Left lateral view of the neurocranium, pectoral girdle and muscles responsible for pectoral girdle adduction in *Cottus perifretum*. **(A)** The different sonic muscles are indicated. **(B)** The protactor pectoralis muscle has been removed. **(C)** The protactor pectoralis muscle, the post-temporal and supracleithrum bones have been removed.

On the basis of movements observed during the high-speed video recordings and the manipulations of freshly dead specimens, dissections mainly focused on the pectoral girdle in order to determine the different osseous structures and muscles that might be involved in sound production (Figures 4,5).

Basically, the skeletal component of the pectoral girdle is composed of three functional units: (1) the shoulder girdle dorsally attaches to the neurocranium; (2) the shoulder plate (i.e. radials, Figure 5A), which is firmly attached to the former element and (3) the fin plate, consisting of fin rays that articulate with the shoulder plate.

Here, we only focus on the shoulder girdle because it is the main skeletal unit that performed noticeable movements when fish produced sounds.

The suspension of the pectoral girdle to the skull occurs through the post-temporal bone (Figure 5). This dermal bone consists of a basal plate with two rostrally directed processes. The dorsal process is thicker and is firmly connected to the epiotic bone *via* a syndesmosis (Figures 4,5). The ventral process is more slender, and extends rostrally into a ligament that inserts on the ventral side of the neurocranium at the level of the otic capsule (Figure 5). Both processes form a fork that aims at restricting rotation around a dorsoventral axis [26,27]. This fork prevents forward displacement of the post-temporal bone, which rotates only slightly. Due to the shape of the epiotic bone, abduction of the pectoral girdle appears to be the most suitable movement.

The supracleithrum is a dermal bone connecting the post-temporal to the cleithral bone. Its lateral face is attached to the medial side of the basal plate of the post-temporal bone, and its ventromedial side is connected to the dorsolateral face of the cleithrum. Such syndesmoses allow some rotation in the plane of the shoulder girdle such as anteroposterior movements.

The cleithral bone constitutes the main part of the shoulder girdle. It suspends all skeletal elements of the pectoral fin and forms the caudal margin of the branchial cavity. Ventrally, the left and right cleithral bones form a symphisis. Dorsally, the cleithral bones of both *Cottus* species display a V-shape formed by two processes (Figure 5C): a rostral and a caudal process. Baudelot’s ligament, which originates on the ventrolateral part of the first vertebra (Figure 4), runs through this V-loop to attach to the lateral face of the cleithrum.

Additionally, dissections highlighted three muscles that might be responsible for the pectoral girdle movements leading to sound production.

The *musculus protactor pectoralis* is the most lateral muscle. It connects the rostral side of the shoulder girdle to the lateral side of the neurocranium at the level of the pterotic bone (Figure 5A).

The *musculus levator pectoralis pars lateralis* (Figures 4,5) originates from the caudal margin of the pterotic bone of the neurocranium. It runs medially to the basal plate of the post-temporal bone, ventrally to the dorsal process and laterally to the ventral process. It inserts on the rostral margin of the cleithral bone.

The *musculus levator pectoralis pars medialis* (Figures 4,5) originates more ventrally to the base of the skull, at the exoccipital bone. Distally, this muscle encloses the ventral process of the post-temporal bone. The fibers are attached to the medial side of the supracleithral bone and the rostral side of the cleithral bone. On the cleithral bone, the insertion of the *pars medialis* is dorsal to the *pars lateralis*, meaning that the two muscles cross each other.

### Morphological characteristics

**Figure 6:**
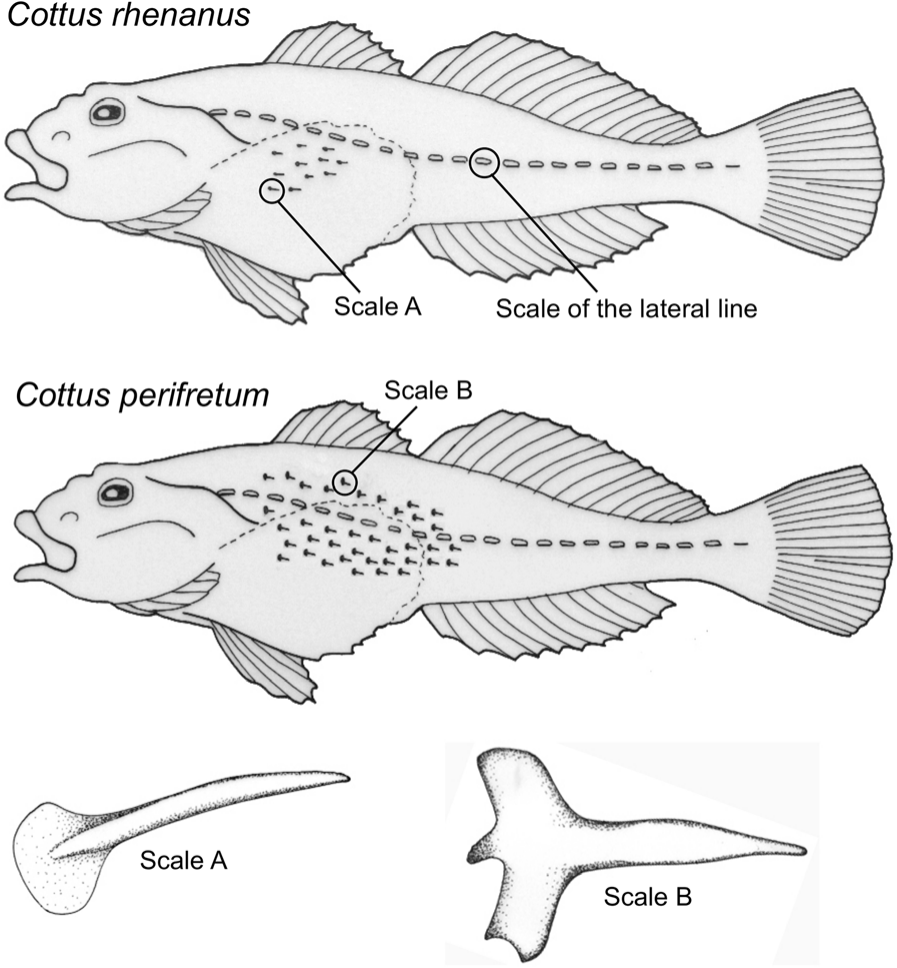
**Depiction of spinelike scales covering the body in *Cottus rhenanus *and *C. perifretum*.** Comparison of the degree to which spinelike scales (prickling) covers the body in both *Cottus* species. Note the striking differences in the shape of prickles between both species*.*

Morphological comparison showed a clear differentiation in spinelike scales (prickling) between *C. rhenanus* and *C. perifretum*. Besides varying in the degree to which skin prickles covers the body (Figure 6, e.g. [3]), another striking diagnostic difference between both sculpin species is the shape of prickles. Indeed, *C. perifretum* possesses more developed spinelike scales with a holly leaf-shaped basis whereas *C. rhenanus* shows prickles with a drop-shaped basis. The spine is also more slender in *C. rhenanus* than in *C. perifretum* (Figure 6).

## Discussion

Although it was previously reported that sound production can occur during both courtship and agonistic behaviors in some *Cottus* species [4,13], calls in *C. rhenanus* and *C. perifretum* were only recorded during agonistic interactions related to territorial defense (an additional audio file shows a multiple-pulsed sound produced by *C. perifretum*; see Additional file 2). Acoustic comparison with other cottid species such as *C. gobio*[10] and *C. paulus*[13] highlights some similarities in sonic features. Generally speaking, sounds produced by these four cottid species exhibit a similar temporal pattern and are relatively low in frequency. Although the average duration of such a call may vary by a factor of two among these four species (125 ms to 265 ms), sound duration in *C. perifretum*, *C. gobio* and *C. paulus* is quite similar and ranges from 229 ms to 264 ms (e.g. [10,13]; see Table 1 for data about *C. perifretum*). Pulse units are also quite similar with a duration varying between 28 ms and 42 ms among all four species (e.g. [10,13]; see Table 1 for data about *C. rhenanus* and *C. perifretum*). Call-dominant frequencies are concentrated between 50 and 500 Hz but *C. rhenanus*, *C. perifretum* and *C. paulus* are contained between 105 Hz and 170 Hz (e.g. [13]; see Table 1 for data about *C. rhenanus* and *C. perifretum*). Another interesting similarity among the different species lies in the sound waveform: the first interpulse duration was always longer, making the first pulse unit in a series distinct from the other ones (Figures 1,2). Although it was not clearly demonstrated in previous studies, the same pattern can be observed for *C. gobio* (see Figure 1 in [10]), and for *C. paulus* (see Figure 2 in [13]).

**Figure 7:**
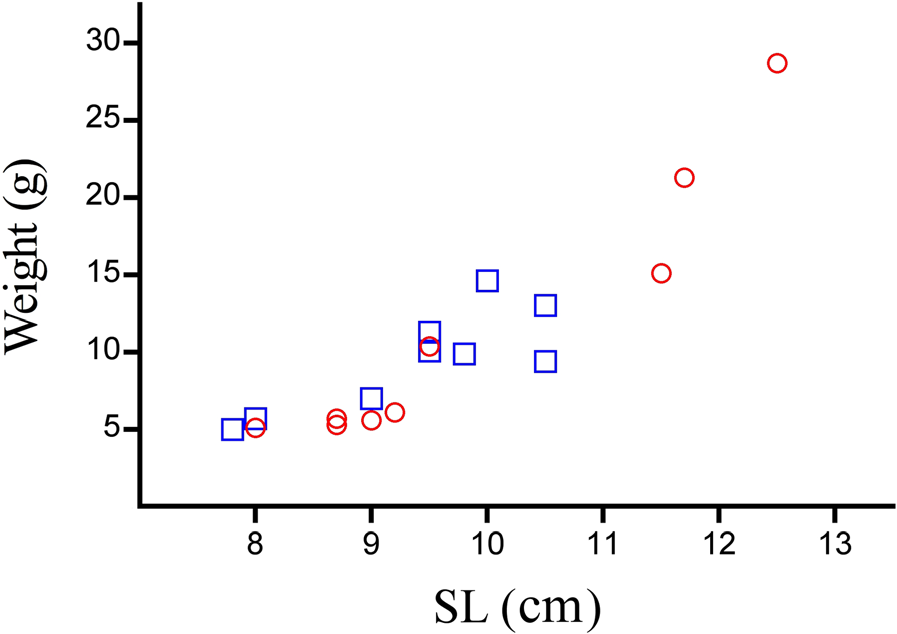
**Size to weight ratio of the two cottid species investigated.** Comparison of the size to weight ratio in the recorded individuals of *Cottus rhenanus* (□) and *Cottus perifretum* (○).

Deeper comparison of agonistic sounds produced by *C. rhenanus* and *C. perifretum* highlights differences in all acoustic features measured (Table 1). Both species differed in pulse duration and dominant frequency but such differences need to be carefully interpreted because these sonic variables can be affected by fish size. The relationships between fish size and both pulse duration and dominant frequency have already been demonstrated for many soniferous fishes [28-36]. Moreover, Ladich [11] noticed that sounds produced by bigger males have lower main frequencies in the river bullhead *C. gobio*. It is likely that differences in pulse duration and dominant frequency between *C. rhenanus* and *C. perifretum* are only due to size differences among recorded individuals, since some specimens of *C. perifretum* were larger (Figure 7). Thereby, these acoustic features cannot be considered as a discriminating character given that no clear size dimorphism was reported between both species [3]. Another difference between both species was observed in sound duration. This can be explained by the differences in pulse duration, and by the differences in the number of pulses per sound. Indeed, the more pulses that occurred per sound, the longer the sound’s duration. The differences observed in the number of pulses per sound might be due to different levels of motivation. Motivation has already been shown to play a role in some teleost fishes during aggressive encounters. In two damselfish species *Dascyllus albisella* and *D. flavicaudus* for example, aggressive sounds are different according to whether they are emitted towards conspecifics or heterospecifics, being multiple-pulsed or single-pulsed, respectively [37,38]. In the river bullhead *C. gobio*, the higher rate of sounds per train was primarily produced by subordinate males, indicating their higher motivation for defending the territory [11]. Thereby, it could be argued that *C. perifretum* produces more pulses per sound than *C. rhenanus* due to a stronger territorial behavior. At last, both species showed differences in the interpulse duration, which is longer in *C. perifretum* than in *C. rhenanus*.

It results from the previous considerations that only the number of pulses per sound and the interpulse duration could be involved in species differentiation. Spanier [39] indicated that these sonic features are the most important parameters for species-specific recognition in four *Stegastes* species (Pomacentridae). Parmentier *et al*. [40] highlighted that four *Dascyllus* species (Pomacentridae) show differences in the interpulse duration. In the same way, Colleye *et al*. [36] argued that differences in the pulse period (i.e. the time interval between two consecutive pulse units in a sound) could help to differentiate cohabiting clownfish species. In this context, it seems that differences in the number of pulses per sound and the interpulse duration could be important for cottid species recognition, especially in the case of *C. rhenanus* and *C. perifretum*, which may come into contact while occurring in sympatry in the lower Rhine drainage.

Regarding auditory sensitivities, our data clearly demonstrate that they do not differ between the investigated species (Figure 3). Hearing sensitivities were characterized based on SPLs and PALs, which is especially important because cottids have no swimbladder and lack hearing specializations. Furthermore, the low absolute threshold (~125 dB re 1 μPa) and low maximum detectable frequency (900 Hz) confirms these fish have poor hearing sensitivity [17]. Interestingly, both species show a clear relationship between sound spectra and their auditory threshold. The average dominant frequencies of acoustic signals is 169 Hz for *C. rhenanus* and 108 Hz for *C. perifretum*, a frequency range close to their best hearing sensitivity at 150 Hz (Figure 3). It is also known that the neuromast hair cells of the lateral line may act as displacement detectors, being stimulated by the particle motion component of sound dominating close to the source (the nearfield), especially for lower frequencies [41,42]. Thus, there exist several reasons to suppose *Cottus* species can hear agonistic sounds produced by their conspecifics: 1) they usually call within 20 cm of the opponent (e.g. [11], pers. obs.), 2) their best hearing sensitivity is at low frequencies (≤ 300 Hz, Figure 3) and, 3) the acoustic signal has already been shown to be in the effective frequency range of the lateral line [16]. By contrast, other observations tend to suggest they could not communicate in their habitat. Ladich [11] reported that the sound pressure level of river bullhead agonistic calls is rather low (110 dB re 1 μPa). Many *Cottus* species are also known to live in a noisy environment (≥ 110 dB re 1 μPa, e.g. [43]). Considering that high amounts of sound energy in these fast-flowing waters are above 1 kHz (leaving a low-energy “noise window” below 1 kHz, e.g. [43]), *Cottus* sounds could be higher than ambient sound level based on their frequencies of vocalization. However, it appears that *Cottus* species produce sounds they could not hear according to our results expressed as pressure threshold (see Figure 3, Table 2). All these findings raise the question over the communicative function of acoustic signals produced during agonistic interactions.

There are several points indicating that sounds might be a by-product of nodding movements associated with pectoral girdle adductions. It has been shown that head nodding movements involve rapid adduction of the pectoral girdle to the cranium [9,10]. While producing agonistic sounds, sculpins rest on the bottom. It is likely that part of the vibrational energy is transferred to the substrate. In the mottled sculpin, Whang & Janssen [12] argued that signals travelling through the substrate are more efficient than sounds travelling through the water because 1) the substrate vibration attenuates at a lesser rate than the water vibration and, 2) the background noise in the substrate, even near riffles that generate much water vibration, is low enough for the fish sounds to be above the noise level. Both the ear and lateral line are also known to be involved in the localization of substrate vibration by the mottled sculpin [20]. Furthermore, the visual component of head nodding might not be regarded as threat display itself. Indeed, sounds usually occur together with a number of more impressive visual threat elements such as raising gill covers, spreading some or all fins, protruding mouth and darkening [10].

All these observations suggest that visual stimuli associated with head nodding might not serve as communication cues. On the other hand, this behavior might be used for conveying useful signals through the substrate due to pectoral girdle vibration.

Although some studies have already focused on the sound-producing mechanism in cottid species, the structure(s) involved in sound production remains unclear. By using electrophysiological techniques in *M. octodecimspinosus*, Barber & Mowbray [9] indicated that sound production results from contractions of specialized muscles originating on the skull and inserting on pectoral girdles. Rapid adductions of the pectoral girdle relative to the skull have also been observed during sound production in *C. gobio*[10], suggesting a similar mechanism of sound production. Based on our dissections of freshly dead specimens in *C. perifretum*, forward displacement of the cleithrum during sound production should be due to the contraction of (at least one) the *levator pectoralis* muscles. This displacement can occur because the osseous fork, formed by ventral and dorsal processes of the post-temporal, forms two anchoring points on the skull, and prevents forward displacements of the post-temporal [26,27]. Electromyography (EMG) experiments conducted in the rock goby *Gobius paganellus* showed the relationship between sound production and contractions of the *levator pectoralis pars medialis* muscle [27]. Cottidae and Gobiidae families are not phylogenetically close [44,45], but both taxa exhibit a very similar morphology, mainly because they have many parallels in their way of life [27]. Contrary to *G. paganellus* for which no pectoral girdle movement has been observed, there is a clear forward displacement of all the shoulder girdle in *C. perifretum* (see Additional file 1), suggesting all the *levator pectoralis* bundles can be involved. Moreover, the crossing insertion of the *levator pectoralis pars medialis* and *pars lateralis* on the cleithral bone should provide a greater resulting force and increase the power of the muscle. However, antagonist muscle (probably the hypaxial muscles) should also participate in the act of sound production because the pulse duration corresponds to a complete back and forth displacement of the pectoral girdle.

There is still a need to provide further insight in identifying the structure responsible for sound production. Observation of the sound waveform highlights that the acoustic pressure of the signal increases steadily, before decreasing in the same way and disappearing into the background noise (Figure 1A,B). Each pulse unit being produced during a complete back and forth movement of the pectoral girdle, sound might result from a process of friction-induced vibrations. Sound production in the channel catfish *Ictalurus punctatus* is based on pectoral girdle mechanism. Hitting its pectoral girdle with a piezoelectric hammer produced sound with a frequency spectrum similar to that of stridulation sounds previously recorded from the same fish [46]. Thus, sounds could be produced by the friction of the Baudelot’s ligament in the V-loop situated on the dorsal part of the cleithrum (Figure 5C), which in turn could set the pectoral girdle into vibration. In addition, the low peak frequency of *Cottus* sounds could be explained by the damping effect attributable to the surrounding muscles (e.g. [47]).

Considering the pectoral girdle as the resonating structure involved in the sound-producing mechanism of cottids could explain the size-related variations observed in pulse duration and dominant frequency. According to Ladich [11], dominant frequency appears to be negatively correlated with fish size in *C. gobio*. A similar correlation has also been observed in the channel catfish *Ictalurus punctatus*[48]. Therefore, a mechanism based on pectoral girdle vibrations might be coherent because sound duration is also strongly predicted by body size in different gobiids [49]. However, further studies are now needed to better understand the resonant properties of the pectoral girdle.

## Conclusions

Overall, it appears that both Belgian *Cottus* species produce agonistic sounds using an acoustic pattern that remained conserved during species diversification. Only the number of pulses per sound and the interpulse duration can be considered as discriminating acoustic features that could be involved in species recognition. However, the acoustic signals do not seem to have a communicative function because fish produce sounds they could not hear in their noisy environment. On the other hand, sounds might be a by-product of nodding movements, and it is likely that fish can detect substrate vibrations further to rapid adductions of the pectoral girdle. Similarities in temporal and spectral characteristics also suggest that Belgian *Cottus* species share a common sound-producing mechanism, likely based on pectoral girdle vibrations. From a morphological point of view, both species are very much alike and they can only be distinguished based on the shape of the prickles covering the body.

## Methods

### Capture and maintenance of fishes

Twenty individuals of *Cottus rhenanus* (total length TL, 78–105 mm) were collected from the Ambleve river (50°23′0.08″N - 5°54′10.24″E, Meuse basin, Belgium) in collaboration with a research team from the LDPH (Laboratory of Fish Demography and Hydroecology, University of Liège, Belgium). Nineteen *Cottus perifretum* (TL, 80–125 mm) were collected from the Tappelbeek stream (49°24′52.2″N - -3°37′2.4″E, Escaut basin, Belgium) in collaboration with a research team from INBO (Instituut voor Natuur- en Bosonderzoek, Linkebeek, Belgium). Both species were caught by electrofishing during October and November 2011, according to the methodology described by Ovidio *et al*. [50]. All specimens were then brought back to the Laboratory of Functional and Evolutionary Morphology (University of Liège, Belgium) where they were transferred to two separate (i.e. one per species) community tanks (1.2 × 0.4 × 0.6 m) filled with running freshwater maintained at 14°C by means of a Ranco air conditioner (type FMI06015854, Germany). These tanks were equipped with a sand bottom, halved flower pots as shelters, and external filters. No internal filters or air stones were used in order to create a quiet acoustic environment for the fishes studied. Fishes were kept under a 12:12 h L:D photoperiod and were fed with red blood worms three times a week.

### Sound recording method

Recordings were made in a smaller glass tank (1.0 × 0.5 × 0.3 m). Following published protocol [10], two males and one female were placed in the centre of the tank equipped with a sand bottom and three halved flower pots, one per male and the last one used as contested territory. The more territories a male has, the better its fitness becomes [5]. Sounds were recorded during territorial defense. After a habituation period of about three days, fishes were recorded in the daytime during one week. Recording sessions lasted approximately 30 min every two hours. Then, fishes were removed and placed back in the community tank, and the process was repeated with three other individuals. Sounds were recorded in 9 *C. rhenanus* (TL, 78–105 mm) and 9 *C. perifretum* (TL, 80–125 mm). Only sounds emitted in series (i.e. multiple-pulsed sounds) were taken into account when conducting sound analysis.

### Sound analysis

Sound recordings were made using a Brüel & Kjaer 8106 hydrophone (Naerum, Denmark, sensitivity: - 173 dB re. 1 V/μPa) connected *via* a Nexus™ conditioning amplifier (type 2690, Naerum, Denmark) to a Tascam HD-P2 stereo audio recorder (Wiesbaden, Germany, recording bandwidth: 20 Hz to 20 kHz ± 1.0 dB). This system has a flat frequency response over wide range between 7 Hz and 80 kHz. The hydrophone was placed just above the three halved flower pots (±5 cm).

Sounds were digitized at 44.1 kHz (16 bit resolution) and analyzed with AviSoft-SAS Lab Pro 4.33 software (1024 point Hanning windowed fast Fourier transform (FFT)). Recording in small tanks induces potential hazards because of reflections and tank resonance [51]. The resonant frequency of the recording tank was determined as 3.01 kHz using a relevant equation from Akamatsu *et al*. [51], and a low pass filter of 3.01 kHz was applied to all sound recordings. Temporal features were measured from the oscillograms whereas peak frequency was obtained from power spectra (filter bandwidth 300 Hz, FFT size point 256, time overlap 96.87% and a flat top window). Generally speaking, the recorded sounds were multiple-pulsed. Hence the following sonic characteristics were measured: number of pulses in a sound, sound duration in ms, pulse duration in ms (the time interval between the onset of one pulse and its end), interpulse duration in ms (the time interval between the end of one pulse and the onset of the next one in a series) and dominant frequency in Hz (frequency component with the most energy).

### Hearing thresholds measurement: experimental setup

Auditory thresholds were determined in 11 *C. rhenanus* (TL, 75–108 mm) and 10 *C. perifretum* (TL, 71–92 mm) by using the auditory evoked potential (AEP) recording technique (e.g. [52]). The AEP technique records far-field potentials in response to sound stimuli of the whole auditory pathway from the inner ear up to midbrain nuclei [53]. Presence or absence of response to sounds of different intensities and frequencies allows the measurement of hearing thresholds. The experimental setup was similar to that used for previous studies [28,54]. Each fish was immobilized in a custom-made harness, restricting body and tail movements while allowing normal respiration. This harness was closed dorsally with small pliers. Three subdermal stainless steel needle electrodes (Rochester Electro-Medical, Lutz, FL, USA) were used for recording the AEP signal. The recording electrode was inserted about 1 mm into the head, over the otic region. The reference electrode was placed within the epaxial musculature, and the ground electrode was placed directly in the water next to the fish. All auditory measurements were carried out in a steel tube (1.2 m high, 22 cm diameter, 0.7 cm thickness) closed at the bottom with a square steel plate (40 × 40 cm) and oriented vertically. The tube was filled with freshwater of approximately 14°C up to a height of 1.12 m. Fishes were suspended 10 cm below the water surface. An UW-30 underwater loudspeaker (Lubell Labs, Colombus, OH, USA) was placed at the bottom of the test tube. The entire setup was enclosed in a walk-in soundproof booth (interior dimensions: 1.8 × 1.8 × 2.1 m).

### Sound stimuli and AEP acquisition

The presentation of sound stimuli and the determination of thresholds followed the detailed description given by Parmentier *et al*. [54]. Both species were tested at six different frequencies: 50, 150, 300, 600, 900 and 1200 Hz. Sound levels at each frequency were presented at up to 164 dB re 1μPa and were attenuated in 6 dB steps until a threshold level was determined. Evoked potentials recorded by the electrode were fed through a TDT HS4-DB4 amplifier (10,000 gain) connected to an RP2.1, routed into the computer and averaged by BioSig software. To measure the evoked response at each level of each frequency, the signal was presented up to a total of 500 times. Acoustic stimuli were calibrated with a Brüel and Kjær 8101 hydrophone (Nærum, Denmark; sensitivity −184 dB re 1 V/μPa; bandwidth 0.1 Hz to 200 kHz) connected to a calibrated Brüel and Kjær 2610 amplifier that gives the absolute sound pressure level. During calibration, the hydrophone was positioned in the experimental setup where the head of the fish had been, and the sound levels were measured with BioSig without phase alternation.

A 4,096-point Fast Fourier Transform (FFT) was used to analyze the AEP signals in the frequency domain. A hearing response was determined to be present if the signal showed the presence of a peak at twice the frequency of the stimulus (e.g. 300 Hz peak when the signal played was 150 Hz), with this peak being at least 3 dB above the background level. The background level was estimated from the AEP power spectrum with a window of 100 Hz around the doubling frequency [24]. Auditory thresholds were defined as the lowest sound level at which significant FFT peaks for the dominant frequency were apparent.

In order to make sure that the AEP signals were not artifacts, we tested our system with dead fishes and with no fish in the experimental setup. No responses were obtained from dead fishes.

### Particle acceleration measurements

In addition SPLs, PALs at thresholds were calculated because it has already been demonstrated that fish species lacking hearing specializations or without swimbladder lack sound pressure sensitivity [17,25,55]. Although we did not possess a calibrated underwater miniature acoustic pressure-acceleration sensor, PALs at all stimulus frequencies and at hearing threshold levels of the fish can be estimated. Basically, particle acceleration was calculated from pressure gradient measurements by a finite difference approximation using the Euler equation [56,57]:

$$a=-\left(\mathrm{grad}\;p/r\right)$$

where *a* is acceleration (m s^-2^); grad *p* the pressure gradient (Pa m^-1^); ρ the water density (0.99924 kg m^-3^ at 14°C).

In pratice, SPLs were recorded at six different locations around the fish’s position (about 10 cm apart in all three orthogonal directions) in the test tube. Consistent with previous studies [24,25,55], the *x*-axis was considered to be the along-body axis (head to tail), the *y*-axis was considered to be the left-right axis on the fish, and the *z*-axis to be the up-down axis. To calculate pressure gradient simply subtract the pressure recording at 2 locations, and divide by the distance. Then divide this pressure gradient by the water density to have an estimate of the accompanying particle acceleration. SPLs were calculated in dB rms re 1 μPa and PALs in dB rms re 1 μm s^-2^.

### Morphological study

Nine *Cottus rhenanus* (TL, 90–105 mm) and nine *C. perifretum* (TL, 95–125 mm) were euthanized with an overdose of MS-222 (500 mg l^–1^) and fixed in 7% buffered formaldehyde for approximately 2 weeks, before being transferred to 70% ethanol for storage. Then, six *C. rhenanus* and five *C. perifretum* were cleared and stained with Alizarin Red S according to Taylor and Van Dyke’s method [58]. These stained specimens were used to look for new morphological characters allowing to discriminate both species. The other individuals were dissected to examine their general morphology using a Leica Wild M10 stereoscopic microscope equipped with a camera lucida and a digital camera (Canon Power Shot S50, Diegem, Belgium). The osteology follows that of Adriaens *et al*. [26] whereas the terminology used for muscles follows that of Winterbottom [59].

### Behavioral observations

While producing sounds during agonistic interactions, fish movements were filmed at 500 fps over three periods of 15 min using an IDT high-speed digital camera NX Series, Model 4-S1 (resolution: 1024 × 1024 pixels, Pasadena, CA, USA). This camera was connected to a laptop (Asus K55V, San Diego, CA, USA) that allowed the visualization of fish movements in real time. This imagery system was also synchronized with a BK 8106 hydrophone connected to a Nexus™ conditioning amplifier type 2690 by means of a Motion Pro Data Acquisition System (Pasadena, CA, USA).

### Statistical analyses

A Shapiro-Wilk test was used to test the normal distribution of acoustic data. As the assumption of normal distribution was not met, a non-parametric Mann–Whitney U test was applied to compare acoustic features between both species. In addition, a non-parametric Friedman’s test with subsequent Dunn’s test for pairwise comparisons was used for both species in order to compare dominant frequencies, pulse durations and interpulse durations within multiple-pulsed sounds.

A non-parametric Mann–Whitney U test was used to compare auditory thresholds expressed in terms of SPLs and PALs between both species for each frequency tested.

All statistical analyses were carried out with STATISTICA 9.1 (StatSoft, Tulsa, OK, USA). Results are presented as means ± S.D. and were considered significant at *P* < 0.05.

## Competing interests

There are not any non-financial competing interests in relation to this manuscript.

## Authors’ contributions

EP designed the study. OC and AS performed sound and AEP recordings, carried out the experimental work and analyzed data. MO provided specific authorizations for collecting specimens in the field. OC wrote the paper with input from EP. All authors read and approved the final manuscript.

## Supplementary Material

Additional file 1 **Behavioral postures exhibited by the river bullhead *Cottus perifretum *** **during sound production.** This movie shows the head nodding movements and pectoral girdle adductions carried out by *Cottus perifretum* while producing multiple-pulsed agonistic sounds. The video was recorded at 500 fps. Click here for file

Additional file 2 **Multiple-pulsed sound produced by *Cottus perifretum *** **during agonistic interactions.** Click here for file
